# Wood Architecture and Composition Are Deeply Remodeled in Frost Sensitive *Eucalyptus* Overexpressing CBF/DREB1 Transcription Factors

**DOI:** 10.3390/ijms21083019

**Published:** 2020-04-24

**Authors:** Phi Bang Cao, Raphaël Ployet, Chien Nguyen, Annabelle Dupas, Nathalie Ladouce, Yves Martinez, Jacqueline Grima-Pettenati, Christiane Marque, Fabien Mounet, Chantal Teulières

**Affiliations:** 1 Laboratoire de Recherche en Sciences Végétales, Université de Toulouse III, CNRS, UPS, UMR 5546, 31320 Castanet-Tolosan, France; phibang.cao@hvu.edu.vn (P.B.C.); raphael.ployet@fabi.up.ac.za (R.P.);; 2 Department of Natural Sciences, Hung Vuong University, Nong Trang Ward, Viet Tri City, Phu Tho Province 29000, Vietnam; 3 Department of Biochemistry, Genetics and Microbiology, Forestry and Agricultural Biotechnology Institute (FABI), University of Pretoria, Pretoria 0002, South Africa; 4 Biotechnology and crop protection Department; Northern Mountainous Agriculture and Forestry Science Institute, Phu Tho 29000, Vietnam; 5 CMEAB, IFR40 Pôle de Biotechnologie Végétale, 31320 Castanet-Tolosan, France

**Keywords:** Wood, CBF/DREB1 transcription factors, transgenic *Eucalyptus*, cold stress, secondary cell walls, lignin, vessels, fibers, wood anatomy

## Abstract

Eucalypts are the most planted trees worldwide, but most of them are frost sensitive. Overexpressing transcription factors for CRT-repeat binding factors (*CBFs*) in transgenic *Eucalyptus* confer cold resistance both in leaves and stems. While wood plays crucial roles in trees and is affected by environmental cues, its potential role in adaptation to cold stress has been neglected. Here, we addressed this question by investigating the changes occurring in wood in response to the overexpression of two *CBFs*, taking advantage of available transgenic *Eucalyptus* lines. We performed histological, biochemical, and transcriptomic analyses on xylem samples. *CBF* ectopic expression led to a reduction of both primary and secondary growth, and triggered changes in xylem architecture with smaller and more frequent vessels and fibers exhibiting reduced lumens. In addition, lignin content and syringyl/guaiacyl (S/G) ratio increased. Consistently, many genes of the phenylpropanoid and lignin branch pathway were upregulated. Most of the features of xylem remodeling induced by *CBF* overexpression are reminiscent of those observed after long exposure of *Eucalyptus* trees to chilling temperatures. Altogether, these results suggest that *CBF* plays a central role in the cross-talk between response to cold and wood formation and that the remodeling of wood is part of the adaptive strategies to face cold stress.

## 1. Introduction

Global climate changes together with an intensification of extreme climatic events are very serious threats to forest trees. Unlike annual herbaceous plants, trees face complex stress conditions repeatedly due to their long life cycle. Among the plethora of negative ecosystemic consequences linked to these climatic changes, a strong decrease in wood production and reduced carbon sequestration are expected [1]. Eucalypts are the most widely planted hardwood forest trees in the world [2] because of their fast growth, superior wood quality, and adaptability to diverse environments. In Europe, eucalypts are mainly planted in Portugal, Spain, Italy, and Southwestern France thanks to favorable climatic conditions for these overwintering evergreen trees. Eucalypt*s* have not evolved avoidance mechanisms such as endodormancy to cope with cold stresses in contrast to deciduous tree species. When temperatures go down in winter, both the primary and secondary (radial) stem growth are temporarily stopped or slowed down but start again when the conditions become favorable [3,4,5]. In eucalypts, the balance between growth and stress resistance is clearly in favor of the former [6].

Secondary growth is the result of the vascular cambium activity that generates secondary xylem (called wood in trees) through a terminal differentiation process, which includes cell division, cell expansion, massive deposition of lignified secondary cell walls (SCWs), programmed cell death, and finally formation of heartwood [7]. Both cambial activity and xylem differentiation are responsive to environmental cues, and wood phenotypic plasticity likely contributes to the functional adaptation of trees to stress [8]. Indeed, vascular traits reflect adaptation to climatic variations in eucalypts grown in their Australian natural environment [9]. In overwintering evergreen trees, xylem vessel anatomy is particularly important since it mediates the trade-off between resistance to freeze–thaw-induced embolism and xylem transport capacity [6]. In addition, changes in the thickness, composition and/or structure of SCWs have been observed in trees such as *Eucalyptus* in response to several stresses such as nitrogen excess or depletion [10], mechanical stress [11], or low temperature [4]. Exposure of *Eucalyptus gunnii* x *dalrympleana* hybrids to long-term chilling temperatures leads to structural modifications of xylem cells, including the deposition of thicker and more lignified xylem cell walls [4]. These results were observed for both young plants grown in controlled conditions and in field-grown adult trees sampled in fall and/or winter.

While we have a relatively good understanding of the developmental regulation of wood formation [12,13], our knowledge of the sensing/signaling pathways and of the mechanisms underlying wood formation in response to stresses is very scarce. Recent studies have highlighted the importance of a dynamic cross-talk between the regulation of SCWs during development and in response to stresses as necessary to promote adaptation to environmental changes. Indeed, some transcription factors (TFs) involved in the regulation of SCW formation are induced by environmental constraints such as high salinity or iron deprivation in Arabidopsis [14] or cold stress in *Eucalyptus* [4]. Notably, some SCW-related TFs are able to confer resistance to salt and drought when ectopically expressed [15,16].

The CRT-repeat binding factor/dehydration responsive element binding1(*CBF*/*DREB1*) genes that belong to the large *AP2/ERF* (APETALA2/ethylene-responsive element binding factor) transcription factor family play key roles in frost tolerance [17]. Indeed, *CBF* overexpression (*CBF*-OE) mimics cold acclimation in addition to developmental and growth modifications in numerous plants species [18,19,20], including *Eucalyptus* [21]. Notably, *CBF* genes are overrepresented in the *E. grandis* genome as compared to most other plant genomes with the exception of *Medicago* that also contains 17 *CBF* members [22]. This subfamily expansion is even more important in *E. gunnii*, which is one of the most frost-tolerant *Eucalyptus* [23]. Some of these *CBFs* are strongly induced in stems in response to cold treatment [23]. To the best of our knowledge, no study has ever investigated the effects of *CBF* overexpression on secondary growth and wood formation.

This prompted us to investigate the effects of *CBF* overexpression on wood formation by using *Eucalyptus CBF*-OE lines that were previously characterized for their tolerance to frost [21]. We analyzed the architecture and the composition of the wood formed in this artificial situation, mimicking permanent cold stress, through histochemical, biochemical, and transcriptomic analyses. The results emphasize the strong impact of *CBF* overexpression on xylem differentiation leading to an altered architecture and composition. The lignin content and composition were also modified as the expression of the main genes involved in lignin biosynthesis.

## 2. Results

Although eucalypts are known to be recalcitrant to transformation, we previously succeeded in transforming a cold-sensitive *Eucalyptus* hybrid (*E. urophylla x E. grandis)* with the two *CBF* genes *EguCBF_K* and *EguCBF_Q* (previously named, respectively, *EguCBF1a* and *EguCBF1b* by [24])*,* cloned from the cold tolerant species *E. gunnii*. The resulting transgenic micro-cuttings overexpressing either *EguCBF_K* or *EguCBF_Q* were shown to be more tolerant to frost and exhibited some phenotypes reminiscent of cold acclimation [21].

As described in the Material and Methods Section, we first implemented a protocol to root the rooting-recalcitrant transgenic micro-cuttings of these two lines *EguCBF_K*-OE and *EguCBF_Q*-OE, and control plants (empty vectors). We then evaluated their tolerance to frost and measured their growth parameters. We also examined in detail the impact of CBF overexpression on xylem formation, architecture, secondary cell wall composition, as well as on the expression of genes involved in the lignin branch pathway.

### 2.1. CBF Overexpression Confers Frost Tolerance to Eucalyptus Transgenic Lines While Negatively Impacting Plant Growth and Development

**Figure 1:**
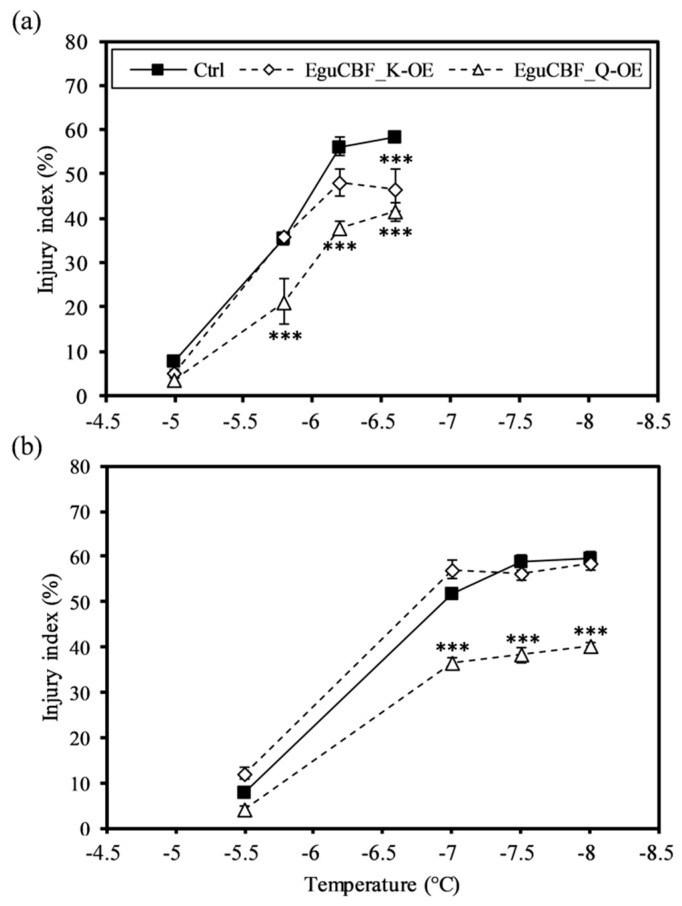
Frost tolerance of CRT-repeat binding factor overexpressing *(CBF*-OE) transgenic lines. Frost tolerance was assessed using the ion leakage method on leaf discs (**a**) and stem segments (**b**) of 6-month-old transgenic plants overexpressing the gene *EguCBF_K* (EguCBF_K-OE) or *EguCBF_Q* (EguCBF_Q-OE) and control plants transformed with an empty vector. Results are means ± SD of three biological replicates. ANOVA and Tukey HSD test *p* < 0.05 (***) were used to highlight statistical differences between transgenic lines and controls.

We assessed frost tolerance on both leaves and stem fragments using the ion leakage method, which allows for the evaluation of cell injury and, therefore, membrane integrity [25]. For all damaging temperatures (below −5.5 °C) (Figure 1), *EguCBF_Q*-OE plants exhibited a significantly lower injury index and thus a better frost tolerance than control lines both for leaves and stems. *EguCBF_K*-OE plants exhibited a significant increase in cold tolerance only for leaf tissues at the lowest temperature assessed, as compared to controls (Figure 1a).

**Figure 2:**
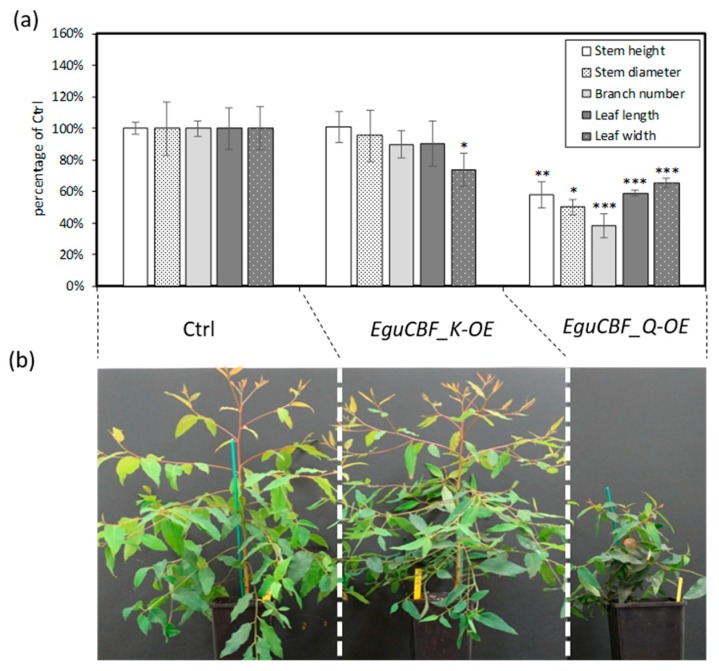
Comparison of morphological parameters between *CBF*-OE and control lines. (**a**) Morphological traits (stem height, stem diameter, branch number, leaf length, and width) were measured on 6-month-old *CBF*-OE (*EguCBF_K*-OE, *EguCBF_Q*-OE) and control plants (Ctrl). The results are means ± SD of six biological replicates. Statistical difference with control was assessed using Tukey HSD test: *** (*p* < 0.001), ** (*p* < 0.01), and * (*p* < 0.05). (**b**) General shape and size of 6-month-old plants.

The higher frost tolerance exhibited by *EguCBF_Q*-OE rooted plants is in good agreement with the results obtained previously on the corresponding micro-cuttings [21] and, notably, was associated with a drastic reduction of primary growth (Figure 2a). In contrast, stem heights of the *EguCBF_K*-OE plants were similar to controls (Figure 2a).

All the morphological parameters measured were strongly decreased (on average by 54%) in *EguCBF_Q*-OE as compared to controls (Figure 2b). Among them, stem diameter and branch number were the most affected, being only 50% and 38% of those of the controls, respectively. In *EguCBF_K*-OE plants, leaf width was significantly reduced while leaf length, number of branches, and stem diameter were slightly but not significantly reduced.

### 2.2. CBF Overexpression Impacts Xylem Differentiation and Architecture in Eucalyptus Transgenic Lines

**Figure 3:**
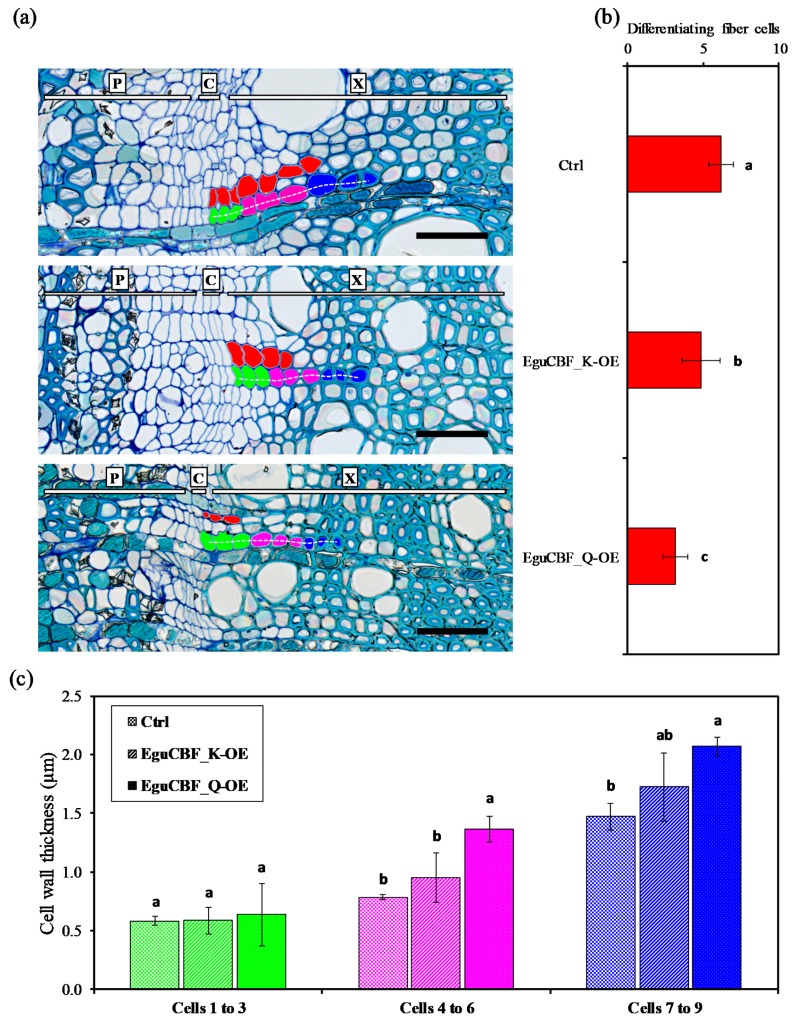
Dynamics of xylem differentiation in the *CBF*-overexpressing lines and controls. (**a**) Stem cross sections of 6-month-old controls, *EguCBF_K*-OE, and *EguCBF_Q*-OE *Eucalyptus* plants stained with Toluidine blue O. Differentiating xylem cells with thin primary cell walls are colored in red. A cell line originating from the same cambial initial was artificially separated into three groups (green, cells 1 to 3; pink, cells 4 to 6; blue, cells 7 to 9) (**b**) Average number of differentiating fiber cells (colored in red) from cambial initials, counted on 49 to 61 fiber cell lines (i.e., fiber cells originating from the same cambial initial) as exemplified by the white dotted line, across 3 biological replicates per line. (**c**) Average cell wall thickness for each of the three groups (cells 1 to 3; 4 to 6; 7 to 9) of differentiating fiber cells starting from the cambial initials. The average cell wall thickness was measured on 31 to 58 fiber cell lines per line, across 3 biological replicates per line. The results are means ± SD of measurements (different fiber cell lines) and statistical difference was assessed using one-way ANOVA and Tukey HSD post hoc test (*p* < 0.05; groups identified with letters a, b and c). P: phloem, C: cambial zone, X: xylem. Scale bar = 50 µm.

The impact of ectopic overexpression of *CBF* on secondary growth was further investigated on stem transversal sections by counting the number of differentiating xylem cells (i.e., xylem cells situated between flat and dividing cambial initials, and xylem cells that initiated secondary cell wall deposition) as a proxy for evaluating cambial activity. The number of differentiating xylem cell layers that exhibited thin primary cell walls (red cells in Figure 3a) was significantly reduced in both *EguCBF_K*-OE and *EguCBF_Q*-OE plants, by 21% and 48%, respectively, as compared to control plants (Figure 3b). We also used cell wall thickness as a proxy for evaluating the xylem cells’ maturation state. For this purpose, we artificially designed three groups of xylem cell layers: 1 to 3 (Figure 3a green), 4 to 6 (Figure 3a pink), and 7 to 9 (Figure 3a blue) adjacent to the cambial initials and measured the average cell wall thickness (Figure 3c). The first three layers 1 to 3 did not exhibit any significant difference between lines as these cells were thin, wall differentiating cells in all lines. In *EguCBF_Q*-OE plants, xylem cells within group 4 to 6 and group 7 to 9 exhibited significantly thicker cell walls than control plants by factors of 1.7 and 1.4, respectively. Although nonsignificant, *EguCBF_K*-OE plants also showed thicker cell walls than controls in these two most inner groups of cells, thus exhibiting an intermediate pattern between controls and *EguCBF_Q*-OE plants. These observations suggest that for both *EguCBF_K*-OE and *EguCBF_Q*-OE, secondary cell wall deposition was initiated earlier than in control plants. This focus on developing xylem from the cambial zone highlighted the significant impact of *CBF* overexpression on cell differentiation and on the dynamics of cell wall deposition.

**Figure 4:**
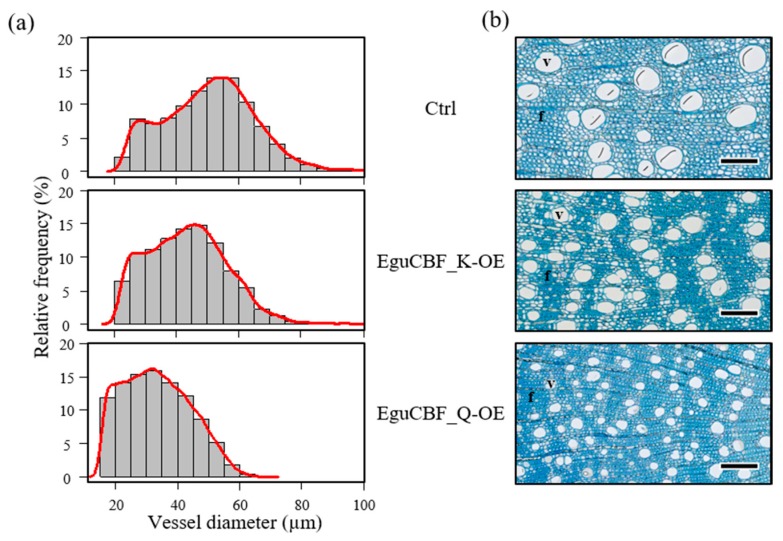
Vessel distribution. (**a**) Vessel diameter distribution in the secondary xylem of 6-month-old control, *EguCBF_K*-OE, and *EguCBF_Q*-OE *Eucalyptus* plants. (**b**) Toluidine blue O stained cross sections of secondary xylem of 6-month-old plants. The results are mean diameters ± SD of a total of 2700 to 6600 vessels measured across 3 biological replicates per line. Scale bar = 50 µm. V: vessels; f: fibers.

We then analyzed the xylem vessels’ properties such as diameter, density, porosity, hydraulic mean diameter, hydraulic conductivity, and vulnerability index. As shown in Figure 4, the distribution of the vessel diameter greatly differed between *EguCBF_Q*-OE plants and controls whereas *EguCBF_K*-OE showed an intermediate pattern. Most of the vessels in *EguCBF_Q*-OE were smaller (71.5% of them showed a maximum diameter of 40 μm), as compared to controls for which 75% of the vessels had a diameter greater than 40 μm. Notably, the smallest vessel diameter class (< 20 μm) was represented only in *EguCBF_Q*-OE, whereas the largest classes (60–80 µm) were virtually absent from *EguCBF_Q*-OE. Concomitant to the reduction of the mean vessel diameter in both *EguCBF_Q*-OE (−34%) and to a lesser extent *EguCBF_K*-OE (−15%; Table 1), the vessel density increased dramatically (+152%) in *EguCBF_Q*-OE and moderately in *EguCBF_K*-OE (+54%). Despite the reduction of their average vessel diameter, secondary xylem of both *EguCBF_K*-OE and *EguCBF_Q*-OE showed an increased porosity, statistically different from controls only for *EguCBF_K*-OE. We then calculated the average vessel hydraulic mean diameter that reflects the class of diameter having the highest contribution to water transport [26]. A similar trend as for the average vessel diameter was observed, where the vessels having the highest contribution to water transport in *EguCBF_K*-OE and *EguCBF_Q*-OE were 14% and 34% smaller, respectively, than in control plants. From these anatomical measurements, we extrapolated the theoretical area-specific hydraulic conductivity known to reflect the efficiency of sap flow through the vascular system [27,28]. It was severely reduced for *EguCBF_Q*-OE (−51%) and less so (−17%) for *EguCBF_K*-OE in comparison to controls. Consequently, the xylem potential vulnerability index was dramatically reduced for both *CBF*-OE lines (−73% and −44% for *EguCBF_Q*-OE and *EguCBF_K*-OE, respectively) as compared to controls, suggesting a lower vulnerability to hydraulic failure for these *CBF*-OE lines [29].

**Figure 5:**
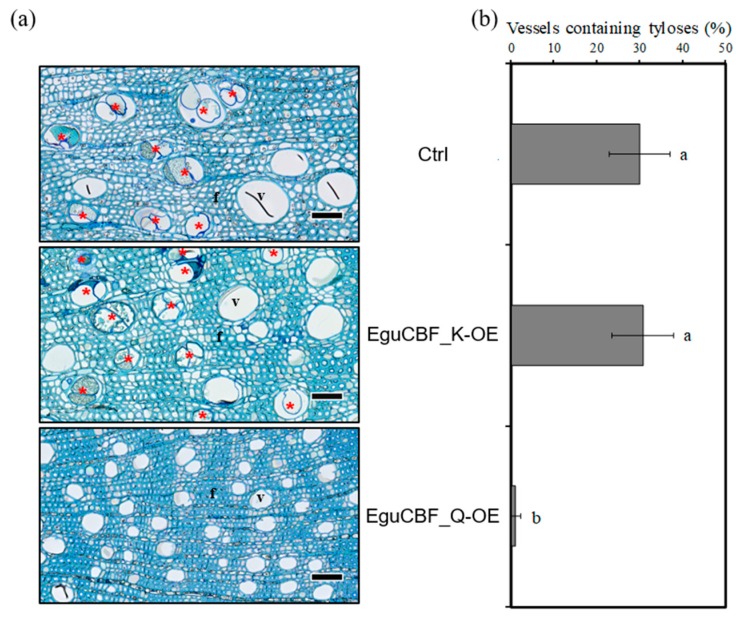
Presence of tyloses in the inner part of the xylem. (**a**) Toluidine blue O stained cross sections of the inner part of the secondary xylem of 6-month-old plants, vessels containing tyloses are highlighted by red stars. (**b**) Percentage of vessels containing tyloses quantified in stem sectors delimitated by ray cells from pith to cambium. A total of 44 to 1915 vessels with tyloses were counted per line, across 3 biological replicates. Statistical significance was assessed with one-way ANOVA and Tukey HSD post hoc test (*p* < 0.05; groups identified with letters a, b and c). Scale bar = 50 µm.

We also quantified the percentage of vessels containing tyloses in their lumen (Figure 5a). Within stem sectors (i.e., areas between two rays, from pith to developing xylem) of controls and *EguCBF_K*-OE plants, on average, 30% of vessels contained tyloses, mainly located in the most mature xylem close to the pith. This percentage was much lower in *EguCBF_Q*-OE (1.1%; Figure 5b).

The characteristics of the fibers of the *CBF*-OE lines were also modified (Table 2). For instance, the fiber lumen diameter was significantly reduced in *EguCBF_Q*-OE plants (−24.3%) and to a lesser extent in *EguCBF_K*-OE plants (8.3%) as compared to controls. The average fiber density showed an opposite trend, with an increase of 7.5% and 26.8% in *EguCBF_K*-OE and *EguCBF_Q*-OE, respectively, as compared to controls. Consistent with these observations, image analyses revealed an overall increase of 8.6% of the cell wall surface proportion in *EguCBF_Q*-OE plants while the average SCW thickness measured over a large number of fibers was not statistically different between the *CBF*-OE lines and the controls.

### 2.3. Both Lignin Content and Composition Are Modified in CBF-Overexpressors

**Figure 6:**
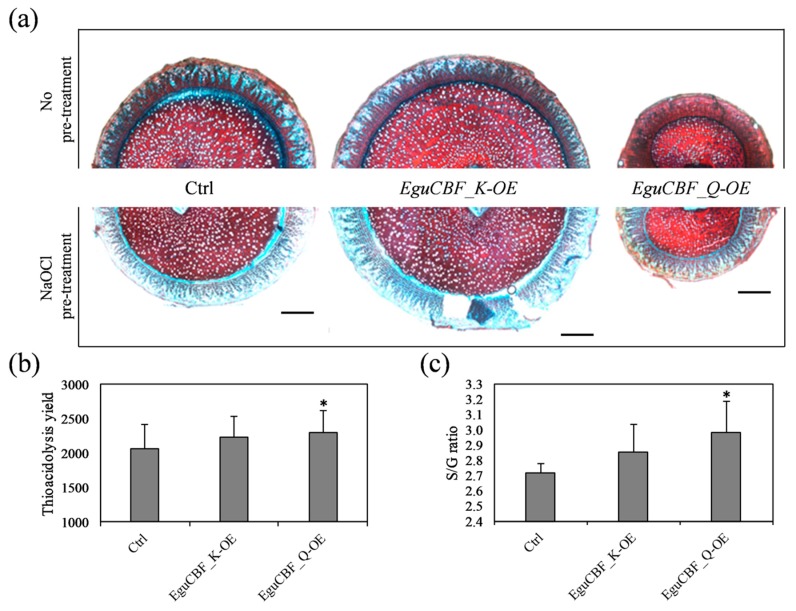
Impact of *CBF* overexpression on xylem biochemical composition. (**a**) Transversal stem sections stained with Safranin–Astra blue counterstaining before (top panel) and after (bottom panel) treatment with sodium hypochlorite (bleaching). (**b**) Total *β*-O-4 linked lignin and (**c**) lignin monomers ratio (S (syringyl)/G (Guaiacyl)) measured through thioacidolysis. Scale bar = 1 mm.

In addition to these changes in wood structure, we observed an accumulation of phenolic compounds using Safranin–Astra blue counterstaining on stem transverse sections (Figure 6a). *EguCBF_Q*-OE plants showed a more intense Safranin red staining than controls in most tissues of the stem (Figure 6a, top panel). This intense staining was considerably attenuated by bleach treatment and washing (Figure 6a, bottom panel), suggesting a strong accumulation of hydrophobic compounds non-covalently bound to the cell wall structures in *EguCBF_Q*-OE plants. The staining profile of *EguCBF_K*-OE stem sections was similar to that of control plants.

To further characterize the modifications occurring in secondary xylem, we analyzed lignin content and structure using thioacidolysis [30], a method that allows quantification of the amount and the composition of the non-condensed *β*-O-4 linked lignin polymer. As shown in Figure 6, the *β*-O-4 lignin content in stems of *EguCBF_Q*-OE plants was significantly increased by 11.8% as compared to controls, whereas *EguCBF_K*-OE showed a nonsignificant increase by 8.6% (Figure 6b). Besides these increases, significant changes were observed in the relative proportion of the S (Syringyl) versu*s* G (Guaiacyl) monomers (Figure 6c). Indeed, the S/G ratio increased from 2.71 (controls) to 2.98 in line-*EguCBF_Q*-OE plants, reflecting either depletion in G monomers and/or enrichment in S monomers within the *β*-O-4 fraction of the lignin polymer. *EguCBF_K*-OE plants showed a nonsignificant increase of the S/G ratio.

### 2.4. Most Lignin Biosynthetic Genes Are Up-Regulated in CBF-Overexpressors

**Figure 7:**
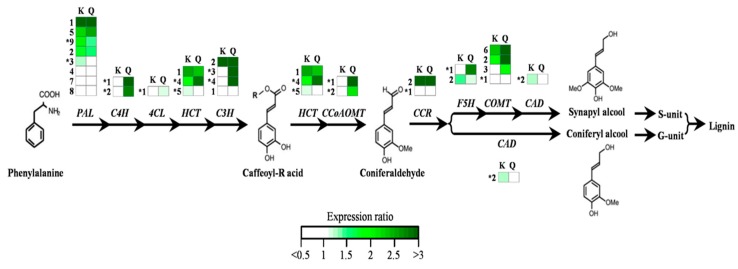
Impact of CBF overexpression on the relative expression of genes involved in the monolignol biosynthetic pathway. Transcript levels are relative to control plants, and were analyzed by RTqPCR. This pathway represents the main biosynthetic route toward the monolignols coniferyl and sinapyl alcohol, giving rise to G and S units, respectively, adapted from [32] (R = shikimic or quinic acid). The analyses included 17 bona fide genes (*) from the core vascular lignin toolbox involved in developmental lignification [31], and 14 additional isoforms showing induction in response to abiotic stresses in previous studies [4]. The green color in the heat maps illustrates induction of gene expression (ratio > 1) in transgenic *CBF*-OE plants, relative to control plants. Ratio values are provided in Table S1. Gene abbreviations are given according to [31]: *PAL*: phenylalanine ammonia lyase; *C4H*: cinnamate-4-hydroxylase; *4CL*: 4-hydroxycinnamate CoA ligase; *HCT*: hydroxycinnamoyl transferase; *C3H*: *p*-coumarate 3-hydroxylase; *CCR*: cinnamoyl CoA reductase; *CCoAOMT*: caffeoyl-CoA 3-*O*-methyltransferase; *F5H*: ferulate 5-hydroxylase; *COMT*: caffeic acid *O*-methyltransferase; *CAD*: cinnamyl alcohol dehydrogenase.

We then evaluated the impact of CBF overexpression on the relative expression of genes encoding enzymes of the different steps of the lignin biosynthesis pathway in *Eucalyptus*. We first analyzed the transcript levels of 15 of the 17 bona fide genes involved in lignin biosynthesis [31] in the secondary xylem of *CBF*-OE plants. In *EguCBF_Q*-OE plants, 10 of these 15 genes were more expressed than in controls with ratios ranging from 1.2 to 3.4 (*p* < 0.05 for 1 gene out of 3 genes tested using Student’s t-test; Figure 7; Table S1). This included genes coding for at least one isoform contributing to each of the early steps of the general phenylpropanoid pathways (*EgrPAL9, EgrC4H1, EgrC4H2, EgrHCT4, EgrC3H3, EgrC3H4, EgrCCoAOMT1, EgrCCoAOMT2*). Consistent with the significant increase of the S/G ratio observed, the gene *EgrF5H1* involved in the biosynthesis of S monolignols was found upregulated by 2.8-fold in *EguCBF_Q*-OE plants, in comparison to control plants. Only 5 out of 15 bona fide genes were more expressed in *EguCBF_K*-OE plants than in controls with ratios ranging from 1.2 to 2.5 (*p* < 0.05 for 1 gene out of 3 genes tested using Student’s t-test; Table S1), mainly involved in the early steps of lignin biosynthesis (*EgrPAL3, EgrPAL9, EgrHCT4, EgrC3H3*) and one gene involved in the final steps of lignin biosynthesis (*EgrCAD2*).

In addition to the core toolbox required for lignin biosynthesis in xylem, we analyzed the expression of 14 genes coding for additional isoforms either contributing to lignin biosynthesis or phenolic compound metabolism in *Eucalyptus* (Figure 7; Table S1; [31]). The majority of these genes had their expression increased in both *CBF*-OE lines, with 9 out of 14 genes upregulated in *EguCBF_Q*-OE by a factor up to 7.1 (significant for 3 genes out of 5 genes tested with Student’s t-test; Table S1), and 9 out of 14 upregulated in *EguCBF_K*-OE plants by a factor up to 3.5 (significant for 4 genes out of 6 genes tested with Student’s t-test; Table S1). Notably, the expression of 7 of these genes (*EgrPAL1, EgrHCT1, EgrC3H2, EgrCCR2, EgrCOMT2, EgrCOMT3,* and *EgrCOMT6*) was already found to be significantly upregulated upon cold exposure in *Eucalyptus* xylem, pointing to a putative role in the stress response [4].

Overall, the induction of at least one isoform for each of the 11 steps of the phenylpropanoid pathways is consistent with the significant increase in lignin content (Figure 6a) and the accumulation of phenolics observed in stem tissues (Figure 6c) of *EguCBF_Q*-OE plants. The phenotype of *EguCBF_K*-OE plants showing neither a significant increase of lignin content nor an apparent accumulation of phenolics is in accordance with the induction of only a few bona fide genes and, overall, only some steps of the phenylpropanoid pathway.

## 3. Discussion

Early work on the *CBF* gene family in *Eucalyptus* allowed for the identification of two *CBF* genes (*EguCBF1a* and *EguCBF1b*) from the cold-tolerant species *E. gunnii* [24,33]. Since then, we characterized the *CBF* gene family in *E. gunnii* from our in-house genome draft [23], and *EguCBF1a* and *EguCBF1b* were renamed *EguCBF_K* and *EguCBF_Q,* respectively. Through characterization of the *CBF* gene family in *E. grandis* [22], we also established putative orthologies between *CBF* members of these two species showing contrasted tolerance to cold [23]. Indeed, *EgrCBF6 (Eucgr.A02824)* and *EgrCBF14 (Eucgr.A02834)* from *E. grandis* were identified as putative orthologs of *EguCBF_K* and *EguCBF_Q*, respectively.

*EguCBF_Q* was shown to be strongly induced in *E. gunnii* stems in response to cold, and *EguCBF_K* was found to be induced in the stem in response to cold to a lesser extent, but with preferential expression in leaves [22,23].

Motivated by the findings that, on the one hand, wood formation is deeply modified by long exposure to chilling temperatures in *Eucalyptus* [4] and, on the other hand, that some *CBF* genes are strongly cold-induced in stems [23], this study aimed at evaluating the role of two of these TFs in controlling wood formation in response to cold.

As previously observed at the micro-cutting stage [21], the phenotyping of rooted six-month-old *CBF*-OE plants revealed an improvement in frost tolerance together with primary and secondary growth reduction, especially dramatic for plants overexpressing *EguCBF_Q*. Here, we showed that *CBF* ectopic expression triggered substantial remodeling of xylem architecture and composition, a phenotype that has never been reported before in any plant species studied.

The vascular phenotype of *CBF*-OE lines included a reduction of xylem vessel diameter together with an increase of vessel density. Reduction of vessel diameter is the most common anatomical wood trait associated with adverse growing conditions such as altitude, frost, or aridity [9,34,35]. Typically observed in angiosperms including *Eucalyptus*, this reduction in vessel surface is compensated for by an increase in vessel density [9].

With regard to fibers, the increase in cell density (i.e., the significant decrease of cell diameter) observed in *CBF*-OE mimics the cold-induced changes observed after long exposure of *Eucalyptus* trees to chilling temperatures [4]. When cambial and differentiating xylem zones were more precisely observed, the number of immature cell layers of the *CBF*-OE was reduced as compared to controls. Such a reduction is commonly reported for deciduous trees during autumnal cold-hardening [36]. This modified dynamic of cell maturation suggests a strong reduction of the cambial activity/expanding zone resembling the dormancy of deciduous trees. Of note, a growing number of reports describe the upregulation of *CBF* in the cambial zone upon dormancy [37], suggesting involvement of *CBF* in the control of cambial activity in trees [34,38,39,40,41,42].

Overall, these *CBF*-OE phenotypes highlighted a deep remodeling of xylem architecture that mostly mimics adaptive features related to plants withstanding stressful environments. The functional significance of the *CBF*-induced changes in *Eucalyptus* wood with regard to stress resistance was supported by both a higher cold tolerance of the stems and a predicted lower vulnerability of the vessels to embolism [35,43]. Beyond the vascular traits, the fiber characteristics of the *CBF*-OE lines may positively influence survival under adverse environmental conditions. The overall increase in cell wall surface as well as the increased lignin content supported by induction of expression of lignin biosynthesis genes as observed in the xylem of *CBF*-overexpressors, may also positively impact cavitation resistance as shown in different species including *Eucalyptus* [44,45]. More generally, these traits likely participate in strengthening the cell walls to prevent freezing damage or cell collapse as reported for cold-hardened plants [46]. Altogether, this data provides evidence that *CBF* overexpression globally promotes the protection of woody organs from frost damage.

The cost of this “safer” phenotype is obviously a clear reduction of growth as observed for *EguCBF_Q-OE* plants. Xylem characteristics may participate in this reduction, since narrow vessels negatively impact hydraulic conductivity [43]. Among overwintering evergreens, *Eucalyptus* is well known for its high hydraulic conductivity associated to a relatively high sensitivity to embolism after freeze–thaw events [6]. In response to constitutive CBF overexpression, which extensively mimics changes associated to adaptation to adverse environmental factors, the response is moved towards safety and reduced hydraulic conductivity. Overall, this *CBF*-OE phenotype highlights the importance of xylem traits in the trade-off between safety (stress resistance) and growth. The presented data also raise the question of the potential involvement of CBF as a potential master player in controlling this compromise.

The present study clearly shows that at least one *Eucalyptus CBF* member (*EguCBF_Q*), which is induced by cold in stems and leads to the most severe modifications of xylem when overexpressed, can control secondary growth in addition to the well-known roles in promoting cold protection and reducing primary growth. By inducing remodeling of anatomy and physiology of both leaves and stems, *CBF* would allow eucalypts to face adverse environments, thereby compensating for the lack of avoidance strategies such as leaf fall and endodormancy.

This novel role for *CBF* in the cross-talk between cold response and wood formation supports that remodeling xylem structure and composition through the *CBF*-signaling pathway is an adaptive strategy to face stress since it is safer to maintain conductivity under stressful conditions. Given that additional *CBF* copies and/or higher expression have been suggested as the main drivers in *E. gunnii* to adapt to a temperate climate [23], the role of potential orthologs of *EguCBF_Q* in *Eucalyptus* genotypes sensitive to frost needs to be addressed.

## 4. Materials and Methods

### 4.1. Plant Material

We previously transformed *E. urophylla x E. grandis* (clone 201) using *A. tumefaciens,* a cold sensitive hybrid, with the coding sequences of two genes *EguCBF1a* and *EguCBF1b* (originated from the cold tolerant *E. gunnii*) under the control of the *35SCaMV* promoter [21]. These sequences were renamed *EguCBF_K* and *EguCBF_Q,* respectively, after annotation of *CBFs* in the genome of *E. gunnii* [23].

We then developed a protocol to root these lines, which were maintained as micro-cuttings by first promoting shoot elongation under low-intensity light (62 µmol/m^−2^/s^−1^) in MS medium [47] supplemented by benzylaminopurine (BAP, 0.5 µM; Sigma, St. Louis, MO, USA; Table S2). Three centimeter long shoots were transferred on 1/4 MS medium supplemented by indol-butyric acid (IBA, 10 µM; Sigma, St. Louis, MO, USA) for three days in the dark at 22 °C to induce root formation. The root elongation phase was conducted in standard conditions for light (16 h/light) and temperature (25 °C-day/22 °C-night) on hormone-free medium (1/4 MS-based) supplemented by charcoal (0.5 g/L; Sigma, St. Louis, MO, USA). After acclimatization on compost supplemented by a fungicide (Propamocarbe fosetyl, 0.3%; Bayer, Leverkusen, Germany) in mini-greenhouses, the rooted plants were grown in a controlled-environment chamber at 25 °C-day/22 °C-night with a long-day photoperiod (16 h/light, 115 µmol/m^−2^/s^−1^ supplied by Lumilux Daylight 58 W Osram, Munich, Germany). Out of 15 independent overexpressing transgenic lines previously characterized for their increased frost tolerance [21], the present study used lines A25 (*EguCBF_K-OE)*, B9 (*EguCBF_Q-OE*) and controls transformed with empty vectors (pK7). After 6 months of ex vitro culture, we measured the main axis height, the stem diameter at the bottom of the main axis, and the branching. We also measured the size (length and width) of 20 mature leaves from 3 plants for each line.

### 4.2. Freezing Tolerance Assessment Using the Ion Leakage Method

From 3 individual plants per line, we collected 8 fresh leaf discs (7 mm diameter) from fully developed leaves (5th rank) and 8 stem fragments (1 cm-long) sampled from the basal part of the plants’ main axes. Samples were transferred into tubes containing 20 mL of de-ionized water and kept at 4 °C overnight. The tubes were transferred into a cryostat (CC2 Huber, Offenburg, Germany) for monitoring the freezing speed (−2.5 °C/h^-1^). At −1 °C, frost was induced by adding an ice cube made from 3 mL of de-ionized water. After freezing at −5 °C, −5.8 °C −6.2 °C, and −6.6 °C for leaf discs and −5.5 °C, −7 °C, −7.5 °C, −8 °C, and −8.5 °C for stem segments, the samples were transferred at 4 °C for thawing slowly overnight. The electrolyte conductivity (Ec) of the solution was measured with a conductance meter (Consort C532, Consort, Turnhout, Belgium) initially (Eci), after thawing (Ecf), and finally after the total cell destruction by autoclaving at 121 °C for 15 min (Ect). The cell membrane injuries were calculated as (Ecf – Eci)/(Ect – Eci) × 100 [48] and expressed as injury indexes (%) representing the susceptibility to frost. Mean values of injury indexes were statistically analyzed by using one-way ANOVA and Tukey HSD post hoc tests implemented in R.

### 4.3. Microscopy and Histochemistry

From the basal parts of plants main axes, stem segments (1 cm-long) were fixed in 3.7% formaldehyde, then dehydrated in 80% ethanol, and a fragment was embedded in LR White resin (London Resin Company Ltd., London, UK). Semi-thin sections (1 μm) were stained with TBO (Toluidine Blue O, Sigma, St. Louis, MO, USA) as previously described [4]. Semi-automatic image acquisition was performed on 3–9 sections (from 3 individual plants per line) in a bright field at 40 × magnification using a Nanozoomer C9600-12 (Hamamatsu, Shizuoka, Japan). The resulting images of whole sections were exported from raw data using NDPview 2.3.1 (Hamamatsu, Shizuoka, Japan) and xylem anatomical characteristics were measured with ImageJ software (V1.5).

Using the particle analysis tool of ImageJ, the lumen cross-sectional area of only functional vessels present in xylem (i.e., vessels without tyloses, diameter ≥ 14μm) was measured automatically. For each transformed line (3 biological replicates), we calculated the average vessel lumen diameter (D, in µm) and the vessel density (N, in mm^−2^) from a total of 2700–6600 vessels. The xylem porosity, defined as the percentage of xylem area occupied by vessels, was inferred from the cumulated area of vessel lumens. The potential xylem vulnerability index (VI = D/N) representing the vulnerability to hydraulic failure was calculated as the ratio between the average vessel diameter (D) and the vessel density (N) as defined by [49]. The theoretical area-specific conductivity (Ks, in Kg.m^−1^/MPa^−1^/s^−1^) of sapwood was extrapolated from these anatomical measurements according to the modified Hagen–Poiseuille equation for capillaries: Ks = (π.ρ/128.η.A).ΣDi^4^, where ρ is the density of water at 20 °C (Kg/m^−3^) and η the dynamic viscosity of water at 20 °C (MPa/s^−1^) [50,51]. The proportion of vessels containing tyloses was calculated as the ratio of vessels showing blue stained material within the lumen, over the total number of vessels, counted manually in a stem sector (from pith to bark) randomly chosen within each section.

Similarly, as for vessels, fibers were automatically detected and their individual lumen cross-sectional areas measured using ImageJ on many representative zones located within the mature xylem and exclusively containing fibers. The average fiber lumen diameter (µm) and the average fiber density (number per mm^2^) were calculated from a total of 4500–19,000 fiber cells measured in 3 biological replicates for each line. The average cell wall thickness of fibers was extrapolated from the total cell wall area, the total fiber lumen area, and the fiber density. Cell wall thickness of differentiating fiber cells was measured on a total of 31–58 fiber cell files across 3 biological replicates per line. Statistical significance of the results was assessed using one-way ANOVA and Tukey HSD post hoc tests implemented in R.

To observe accumulation of hydrophobic compounds, 60-μm-thick stem transverse sections were stained with Astra blue and counterstained with Safranin (Sigma, St. Louis, MO, USA). Sections stored in 80% ethanol were first rehydrated for 10 min in distilled water, pretreated or not with 1% (*v*/*v*) sodium hypochlorite solution, and then immersed successively for 10 min in 1% (*m*/*v*) of aqueous Astra blue, and in 0.1% (*m*/*v*) aqueous Safranin, with an intermediate washing step in distilled water.

### 4.4. Lignin Content and Monomer Composition

Bark-free samples from the basal parts of the plant main stem axes were oven-dried for 48 h at 60 °C and then ground to powder. Lignin content and structure were evaluated using the simplified thioacidolysis method described by Méchin et al. [30] using 10 mg of pre-extracted tissues as described in Ployet et al. [4].

### 4.5. RNA Isolation and RT-qPCR Analysis

The transcript quantification of the *CBF* transgene and of genes encoding phenylpropanoid pathway genes previously identified in *Eucalyptus* [22,31] was completed as follows. Total RNA was extracted from 50 mg of frozen samples (leaves or stems) from 3 biological replicates per line using the CTAB-based protocol and following DNAse treatment using Ambion^®^ DNase I (Thermo Fisher, Waltham, MA, United States). cDNA was produced from 3µg of RNA, as previously described [23]. The RT-qPCR reactions were performed using 1 µL of 1/10 diluted cDNA template and Applied Biosystems^®^ Power SYBR^®^ Green PCR Master Mix ( Thermo Fisher, Waltham, MA, United States), as described previously [52]. Primers used in this study were previously designed [31] and are listed in Table S3. The changes in gene expression relative to control plants were quantified using the 2-ΔΔCt method [53] using five reference genes [52] (Table S1), and statistically assessed using Student’s t-test when the number of biological replicates per line was higher or equal to three.

## Figures and Tables

**Table 1:** Vessel properties in *Eucalyptus CBF*-overexpressors.

	Control	*EguCBF_K*-OE	*EguCBF_Q*-OE
Vessel diameter (µm)	52.7 ± 3.4 ^a^	44.9 ± 4.3 ^b^	34.6 ± 5.4 ^c^
Vessel density (N/mm^−2^)	67.4 ± 10.8 ^c^	103.6 ± 18.2 ^b^	169.9 ± 39.5 ^a^
Porosity (%)	14.5 ± 1.4 ^b^	16.1 ± 1.5 ^a^	15.4 ± 2.6 ^ab^
Average vessel hydraulic mean diameter (µm)	62.8 ± 4.7 ^a^	54.2 ± 6.6 ^b^	41.4 ± 6.4 ^c^
Theoretical area-specific hydraulic conductivity (kg/m^−1^/MPa^−1^/s^−1^)	15.8 ± 2.9 ^a^	13.1 ± 3.6 ^b^	7.7 ± 3.2 ^c^
Potential vulnerability index	0.81 ± 0.16 ^a^	0.45 ± 0.11 ^b^	0.22 ± 0.08 ^c^

These parameters were evaluated on 2700–6600 vessels from 3 replicates for each line. Statistical significance was assessed through two ways ANOVA and groups (identified with letters a, b and c) were defined using Tukey post hoc test (*p* < 0.05).

**Table 2:** Properties of xylem fibers in *Eucalyptus CBF*-overexpressors.

	Control	*EguCBF_K*-OE	*EguCBF_Q*-OE
Fiber lumen diameter (µm)	5.51 ± 0.62 ^a^	5.05 ± 0.74 ^b^	4.17 ± 0.41 ^c^
Fiber density (N/mm^−2^)	9793.1 ± 1639.2 ^c^	10594.6 ± 1684.9 ^b^	13378.7 ± 2214.1 ^a^
Fiber cell wall thickness (µm)	3.00 ± 0.49 ^a^	3.01 ± 0.70 ^a^	2.85 ± 0.47 ^a^
Cell wall area fraction (%)	58.19 ± 2.99 ^b^	56.66 ± 5.37 ^b^	63.19 ± 5.21 ^a^

The fiber lumen diameter and density are the average of 4500–19,000 measurements across 3 biological replicates per line. The cell wall thickness is the average of measurements performed on 38,000–65,000 fibers across 3 biological replicates per line. Statistical significance was assessed through two ways ANOVA and groups defined using Tukey HSD post hoc test (*p* < 0.05; groups identified with letters a, b and c).

## Supplementary Materials

Supplementary Materials can be found at https://www.mdpi.com/1422-0067/21/8/3019/s1. Table S1: Expression ratios and results of statistical tests performed on genes related to monolignol biosynthesis pathway in CBF-OE transgenic lines and controls; Table S2: Composition of MS medium (W4) used for promoting shoot elongation in propagation of transgenic and control lines; Table S3: List of primers used for analysis of transcript level of lignin-related genes by RT-qPCR.

## Abbreviations

4CL	4-hydroxycinnamate CoA ligase
ANOVA	Analysis of variance
AP2/ERF	APETALA2/ethylene-responsive element binding factor
BAP	Benzylaminopurine
C3H	*p*-coumarate 3-hydroxylase
C4H	Cinnamate-4-hydroxylase
CAD	Cinnamyl alcohol dehydrogenase
CaMV	Cauliflower mosaic virus
CBF	CRT-repeat binding factors
DREB	Dehydration responsive element binding protein
CCoAOMT	Caffeoyl-CoA 3-*O*-methyltransferase
CCR	Cinnamoyl-CoA reductase
cDNA	Complementary DNA
COMT	Caffeic acid *O*-methyltransferase
CTAB	Cetyltrimethylammonium bromide
Ec	Electrolyte conductivity
F5H	Ferulate 5-hydroxylase
G	Guaiacyl
HCT	Hydroxycinnamoyl transferase
HSD	Honestly significant difference
IBA	Indol-butyric acid
OE	Overexpression
PAL	Phenylalanine ammonialyase
PCR	Polymerase chain reaction
RTqPCR	Real time quantitative polymerase chain reaction
S	Syringyl
SCW	Secondary cell wall
TBO	Toluidine blue O
TF	Transcription factor
VI	Vulnerability index
