# Supplementary material for: Wood Architecture and Composition Are Deeply Remodeled in Frost Sensitive Eucalyptus Overexpressing CBF/DREB1 Transcription Factors
Source: Int J Mol Sci. 2020 Apr 24;21(8):3019. doi: 10.3390/ijms21083019 (PMC7215815; doi:10.3390/ijms21083019)
Supplement: Supplementary file 1 [file ijms-21-03019-s001.pdf]

International Journal of
Molecular Sciences

MDPI

Supplementary Materials: Wood Architecture and Composition Are Deeply Remodeled in Frost Sensitive *Eucalyptus* Overexpressing CBF/DREB1 Transcription Factors

Phi Bang Cao, Raphaël Ployet, Chien Nguyen, Annabelle Dupas, Nathalie Ladouce, Yves Martinez, Jacqueline Grima-Pettenati, Christiane Marque, Fabien Mounet and Chantal Teulières

International Journal of *Molecular Sciences*

MDPI

Table S1. Expression of genes related to monolignol biosynthesis pathway in *CBF*-OE transgenic *Eucalyptus*.

Gene Family (*Bona Fide* Analysed/Total)	Gene Alias (* *Bona Fide*)	Gene Accession	Student's T test Performed	EguCBF_K-OE	Significant (T test *P* value)	EguCBF_Q-OE	Significant (T test *P* value)	Cold Responsive Ployet et al., 2017
PAL (2/2)	EgrPAL1	Eucgr.A01144	+	**2.97**		**4.78**	*P* < 0.05	+
	EgrPAL2	Eucgr.C03570	+	**1.77**	*P* < 0.05	**1.53**		
	****EgrPAL3***	Eucgr.G02848	+	**1.22**		0.59		+
	EgrPAL4	Eucgr.G02849	+	1.1		0.86		
	EgrPAL5	Eucgr.G02850	+	**2.21**	*P* < 0.05	**2.55**	*P* < 0.05	
	EgrPAL7	Eucgr.G02852	+	1.1		0.83		
	EgrPAL8	Eucgr.J00907	+	0.81		0.87		+
	****EgrPAL9***	Eucgr.J01079	+	**2.09**	*P* < 0.05	**1.49**		+
C4H (2/2)	****EgrC4H2***	Eucgr.C00065		0.58		**2.79**		+
	****EgrC4H1***	Eucgr.J01844		0.75		**3.4**		+
4CL (1/1)	****Egr4CL1***	Eucgr.C02284	+	1.02		1.18		+
HCT (2/2)	EgrHCT1	Eucgr.F03972		**2.6**		**2.38**		+
	****EgrHCT4***	Eucgr.F03978		**1.94**		**2.9**		
	****EgrHCT5***	Eucgr.J03126	+	1.14		0.67		+
C3H (2/2)	EgrC3H1	Eucgr.A02185	+	0.85		0.43		+
	EgrC3H2	Eucgr.A02188	+	**2.66**	*P*<0.05	**2.45**		+
	****EgrC3H3***	Eucgr.A02190		**2.53**		**3.2**		+
	****EgrC3H4***	Eucgr.G03199	+	0.64		**2.99**	*P* < 0.05	+
CCoAOMT (2/2)	****EgrCCoAOMT1***	Eucgr.I01134		0.9		**3.07**		+
	****EgrCCoAOMT2***	Eucgr.G01417		0.96		**2.2**		+
CCR (1/1)	****EgrCCR1***	Eucgr.J03114	+	0.87		0.79		+
	EgrCCR2	Eucgr.F03954	+	**3.46**	*P* < 0.05	**7.13**	*P* < 0.05	+
F5H (1/1)	EgrF5H2	Eucgr.I02371	+	**1.45**		1.12		
	****EgrF5H1***	Eucgr.J02393		1		**2.77**		

COMT (1/1)	***EgrCOMT1**	Eucgr.A01397	+	0.93	0.61	
	EgrCOMT2	Eucgr.A01389		**1.95**	**3.7**	+
	EgrCOMT3	Eucgr.A01392		0.56	**2**	+
	EgrCOMT6	Eucgr.K00041		**2.38**	**3.82**	+
CAD (1/2)	***EgrCAD2**	Eucgr.G01350	+	**1.22**	1	+

Expression values are ratios of average transcript level detected in 3 biological replicates per line, relative to control transgenic plants. Genes short names are according to [32]: PAL, phenylalanine ammonialyase; C4H, Cinnamate-4-hydroxylase; 4CL, 4-hydroxycinnamate CoA ligase; HCT, hydroxycinnamoyl transferase; C3H, p-coumarate 3-hydroxylase; CCR, cinnamoyl CoA reductase; CCoAOMT, caffeoyl-CoA 3-O-methyltransferase; F5H, ferulate 5-hydroxylase; COMT, caffeic acid O-methyltransferase; CAD, cinnamyl alcohol dehydrogenase. In bold and identified with (*) are the *bona fide* genes belonging to the lignin toolbox in *Eucalyptus* [32]. Ratios higher than 1.2 are highlighted in bold. Statistical significance was assessed for 18 genes using Student's T test on 3 to 6 biological replicates.

International Journal of *Molecular Sciences*

MDPI

Table S2. Composition of MS medium (W4) promoting shoot elongation.

**Macroelements (mg. L^{1})**		**Fe-EDTA (mg. L^{1})**	
NH_4NO_3	1650	$FeSO_4$. $7H_2O$	27.85
KNO_3	1900	Na_2EDTA	37.25
$CaCl_2.2H_2O$	440	**Vitamins (mg.L^{1})**	
$MgSO_4$. $7H_2O$	370	Nicotinic acid	0.1
KH_2PO_4	170	Thiamine-HCl	0.5
**Oligoelements (mg.L^{1})**		Pyridoxine-HCl	0.1
$MnSO_4$. H_2O	16.9	Myo-inositol	100
$ZnSO_4$. $7H_2O$	10.6	**Saccharose (g. L^{1})**	20
H_3BO_3	6.2	**Phytagel (g. L^{-1})**	3.5
KI	0.83	**Hormones (µM)**	
Na_2MoO_4. $2H_2O$	0.25	BAP*	0.5
$CuSO_4$. $5H_2O$	0.025		
$CoCl_2.6H_2O$	0.025	**pH**	6

(*) Added after sterilization.

Table S3. List of oligonucleotide sequences used as primers for RT-qPCR reactions.

Gene Name	Accession Number	Forward Primers	Reverse Primers
**Transgenes**			
EguCBF_K-OE		GTACAAAAAAGCAGGCTATATGAACC C	TGGCACTCCTTCCGTCAGAT
EguCBF_Q-OE		CCAGAGAGGACAACGGGAAC	GAAAGCTGGGTAACATGGAATAGCTC
**Genes Involved in Lignin Biosynthesis**			
EgrPAL1	Eucgr.A01144	TCAAGGATTGCGGGTCGTATCC	ACCCTTTCTCCGGTCAGCAAAC
EgrPAL2	Eucgr.C03570	GATGGCTTGCTGCTTGAATCGC	AGACGGGCTCATGTTCAACGAC
EgrPAL3	Eucgr.G02848	TGGGTGATGGAGAGCATGAGCAAG	ATGCCGGCGTTCAAGAACCTTATG
EgrPAL4	Eucgr.G02849	CAGACGGAGCTCATAAGGTTCTTG	GTGTTGATCCTCACGAGCATGG
EgrPAL5	Eucgr.G02850	GCGCATGTGAATTTCCTTCATAGC	ATCGAGCGAGGATCAAGGTGAG
EgrPAL7	Eucgr.G02852	GGCATATTCGGCAATGGGACTG	TGTTGATCCTCACGAGCATTGCG
EgrPAL8	Eucgr.J00907	GGAGTGGATCATGGAGAGCATGTG	ACACCGGCGTTCAAGAATCTGATG
EgrPAL9	Eucgr.J01079	TGGAAGGAGCCAGAATCATGCC	TTGTGGACCGACCATAATGTGC
EgrC4H1	Eucgr.J01844	TGAGAAGGGTGGCCAATTCAGC	TGGCTTCAAGACGATGGTGGAG
EgrC4H2	Eucgr.C00065	ACTGTCGTCTTTGAGCCCGTTG	GGCTGCCCATTTGGTTTAATGCG
Egr4CL1	Eucgr.C02284	AGGAACGCGGAGATGAAGATCG	CGGCGTCGTTCAGATAACCTTTC
EgrHCT1	Eucgr.F03972	CCATTGAAGCTCGCAGTCTAGC	GATATCATCCGAGTGCCCTTATGC
EgrHCT4	Eucgr.F03978	GCGCATGTTGTTCATTCTTCATGC	GCAATGCATCCAATCCCACTGC
EgrHCT5	Eucgr.J03126	CGGAAGCTCATTCCAGCTGTAGAC	TTGAAGTACGTCACCTGCAACAC
EgrC3H1	Eucgr.A02185	CGGCAACCTCTACGACATCAAATC	AGCTTGACACGACCACGTTCAG
EgrC3H2	Eucgr.A02188	ACGTGAAGGTCAGGAAGGTGTG	GCACGGTCTTCCTCGAATTATCGG
EgrC3H3	Eucgr.A02190	TGCACCAACCCTGATAATTCGG	CGCCAGCCTCGTTATGTTGTTG
EgrC3H4	Eucgr.G03199	GGAAGTTATGACCTGAGCGAGGAC	GCCAGTGATCATGTCCCAAAGG
EgrCCoAOMT1	Eucgr.I01134	AGAAGGCCCTGCTTTGCCTATTC	AACGACCCTTGCTTCCCATCTTC
EgrCCoAOMT2	Eucgr.G01417	ATCAGCTCGTGCAAGATGAGAAG	TGTAGTTGTCCTTGTCGGCATCC
EgrCCR1	Eucgr.J03114	GCGATGTGGTGGAAATCCTTGC	GGGTTCACCTCATCAGAGCACTTG
EgrCCR2	Eucgr.F03954	AGAGGTTGACTCTGTGCAAGGC	GCTGGCTCCAACATTTGCTCTC
EgrF5H1	Eucgr.J02393	AAGCAAATGGAGGGTCGGGTTG	TCCAAATCTTGCAGCCCTCCTG
EgrF5H2	Eucgr.I02371	ATTGAGACGAGGCATCGAACCG	CGCATGCAAAGCCAGCCATTTC
EgrCOMT1	Eucgr.A01397	CACGTGATTGAAGACGCTCCAC	CTCCAGTCATGGCATATCCACTTC
EgrCOMT2	Eucgr.A01389	ACAGCTTGAACTGTGCTCTCCTG	AAGGCTCATTTGCGACCTATCG
EgrCOMT3	Eucgr.A01392	AGACGATGGTTGATGTTGGAGGTG	AACGTGTTCCACACCGGGATAG
EgrCOMT6	Eucgr.K00041	CTGATCATGATGGCGCACAACC	CAAAGGCAACGCACGCGATATG
EgrCAD2	Eucgr.G01350	AGTCCCGCTTAGGTTAGCTGTC	AAAGAGAAATCCACTTCCGCTGTC
**Reference Genes**			
EgrSAND	Eucgr.B02502	TTGATCCACTTGCGGACAAGGC	TCACCCATTGACATACACGATTGC
EgrEF-1a	Eucgr.B02473	ATGCGTCAGACTGTGGCTGTTG	TTGGTCACCTTGGCTCCACTTG
EgrIDH	Eucgr.F02901	AATCGACCTGCTTCGACCCTTC	TCGACCTTGATCTTCTCGAAACCC
EgrPP2A1	Eucgr.B03386	TCGAGCTTTGGACCGCATACAAG	ACCACAAGAGGTCACACATTGGC
EgrPP2A3	Eucgr.B03031	CAGCGGCAAACAACTTGAAGCG	ATTATGTGCTGCATTGCCCAGTC
